# DADS Suppresses Human Esophageal Xenograft Tumors through RAF/MEK/ERK and Mitochondria-Dependent Pathways

**DOI:** 10.3390/ijms150712422

**Published:** 2014-07-14

**Authors:** Xiaoran Yin, Jun Zhang, Xiaoning Li, Dong Liu, Cheng Feng, Rongrui Liang, Kun Zhuang, Chenlei Cai, Xinghuan Xue, Fuchun Jing, Xijing Wang, Jun Wang, Xinlian Liu, Hongbing Ma

**Affiliations:** 1 Department of Oncology, the Second Affiliated Hospital of Xi’an Jiaotong University, Xi’an 710004, China; E-Mails: yinxiaoran@163.com (X.Y.); qingcao0066@163.com (Xia.L.); xuexinghuan1962@163.com (X.X.); wangxijingxian@126.com (X.W.); liuxldoc@yeah.net (Xin.L.); 2 Department of Digestion, the Second Affiliated Hospital of Xi’an Jiaotong University, Xi’an 710004, China; E-Mails: jun3z@163.com (J.Z.); liudong_doc@163.com (D.L.); fc_2004188@163.com (C.F.); cchenlei2007@163.com (C.C.); 3 Department of Oncology, the First Affiliated Hospital of Soochow University, No. 188, Shizi Street, Suzhou 215006, China; E-Mail: lengbeng@hotmail.com; 4 Department of Digestion, Xi’an Central Hospital, Xi’an 710003, China; E-Mail: zhuanger6695@sohu.com; 5 Department of Digestive Diseases, Baoji People’s Hospital, Baoji 721000, China; E-Mail: fleming7798@163.com; 6 Department of Gastroenterology, Xi’an Hospital of Traditional Chinese Medicine, Xi’an 710001, China; E-Mail: wangjuntaiji@126.com

**Keywords:** esophageal carcinoma, DADS, apoptosis, animal model

## Abstract

Diallyl disulfide (DADS) is a natural organosulfur compound isolated from garlic. DADS has various biological properties, including anticancer, antiangiogenic, and antioxidant effects. However, the anticancer mechanisms of DADS in human esophageal carcinoma have not been elucidated, especially *in vivo*. In this study, MTT assay showed that DADS significantly reduced cell viability in human esophageal carcinoma ECA109 cells, but was relatively less toxic in normal liver cells. The pro–apoptotic effect of DADS on ECA109 cells was detected by Annexin V-FITC/propidium iodide (PI) staining. Flow cytometry analysis showed that DADS promoted apoptosis in a dose-dependent manner and the apoptosis rate could be decreased by caspase-3 inhibitor Ac-DEVD-CHO. Xenograft study in nude mice showed that DADS treatment inhibited the growth of ECA109 tumor in both 20 and 40 mg/kg DADS groups without obvious side effects. DADS inhibited ECA109 tumor proliferation by down-regulating proliferation cell nuclear antigen (PCNA). DADS induced apoptosis by activating a mitochondria-dependent pathway with the executor of caspase-3, increasing p53 level and Bax/Bcl-2 ratio, and downregulating the RAF/MEK/ERK pathway in ECA109 xenograft tumosr. Based on studies in cell culture and animal models, the findings here indicate that DADS is an effective and safe anti-cancer agent for esophageal carcinoma.

## 1. Introduction

Esophageal carcinoma is among the most common tumors in the world, ranking eighth in occurrence and sixth in mortality [1]. Approximately 70% of global esophageal carcinoma cases occur in China and the five–year survival rate is only 10% [2]. Generally, esophageal carcinoma patients in the primary stage can be cured by surgical resection. However, a majority of patients in the advanced stage eventually succumb to this disease [3]. Although surgery, radiotherapy and chemotherapy are regarded as important parts of the systemic therapy for metastatic esophageal carcinoma, the success of such treatments have been weakened by their severe systemic toxicities and local irritating effects. Therefore, in order to enhance efficacy and reduce toxicity, researchers have urgently tried to develop novel regimens with fewer side effects against esophageal carcinoma [4].

Plant-derived herbal medicines have been used in several Asian countries including China for a long time. Some plant compounds have anticancer activity with low toxicity and could be used as alternative chemotherapeutic agents for carcinomas [5]. Garlic, as an herbal medicine, has antimicrobial, antiplatelet, antithrombotic, antiarthritic and antitumorigenic properties [6]. Diallyl disulfide (DADS), CH_2_=CH–CH_2_–S–S–CH_2_CH=CH_2_, is a lipid–soluble organic compound isolated from garlic. Scientific investigations have shown that DADS reduces the risk of cardiovascular disease and diabetes [7], serves as anti-oxidant [8], fights against infections [9], and exhibits significant protection effects against malignancies [10,11,12]. Moreover, in human breast cancer MCF–7 cells, the apoptotic effect of DADS is even superior to chemotherapy agents such as 5F-dUMP (5-Fu) and cyclophosphamide (CTX) [13].

Considering limited data on DADS bioavailability, additional studies are warranted to check the effects of DADS in animal models of esophageal carcinoma [11,14]. Moreover, the molecular mechanisms of DADS on various carcinomas are still a matter of debate and the exact effects of DADS *in vivo* on esophageal carcinoma are still unclear. Therefore, in the present study, we used an ECA109 xenograft model in nude mice to study the effect of DADS on tumor growth. Additionally, we investigated the potential biomarkers and associated molecular alterations in ECA109 xenograft tumor. This is the first study to evaluate the potential effects and mechanisms of DADS on human esophageal carcinoma *in vivo*.

## 2. Results and Discussion

### 2.1. Diallyl Disulfide (DADS) Inhibits Cell Viability

**Figure 1:**
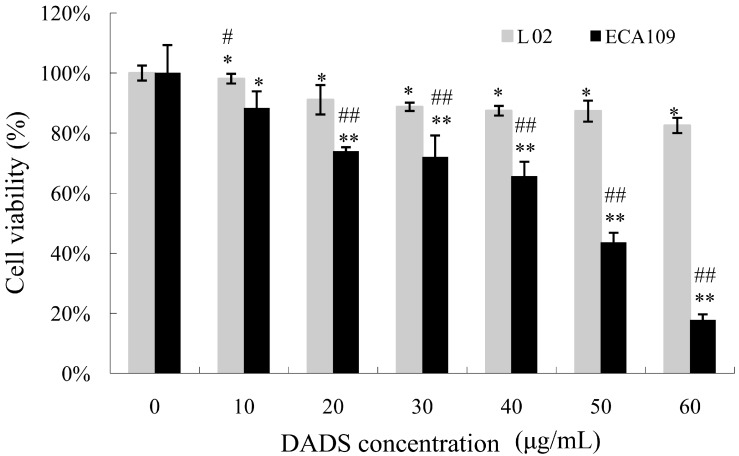
Viability inhibitory effects of diallyl disulfide (DADS) on ECA109 cells and L02 cells. ECA109 and L02 cells were incubated with different doses of DADS (0, 10, 20, 30, 40, 50, 60 μg/mL) for 24 h respectively. Cell viability was detected by MTT assay and was represented as the percentage of relative absorbance. Data are expressed as the mean ± SD from five independent experiments (* *p <* 0.05, ** *p <* 0.01 compared with the control group, # *p <* 0.05, ## *p <* 0.01 ECA109 compared with L02).

**Figure 2:**
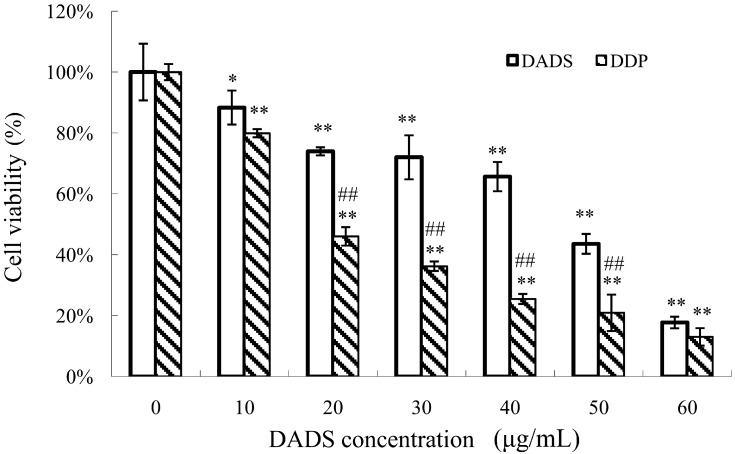
Viability inhibitory effects of DADS and *cis*-diammminedichloroplatinum (DDP) on ECA109 cells. ECA109 cells were incubated with different doses of DADS and DDP (0, 10, 20, 30, 40, 50, 60 μg/mL) for 24 h respectively. Cell viability was detected by MTT assay and was represented as the percentage of relative absorbance. Data are expressed as the mean ± SD from five independent experiments (* *p <* 0.05, ** *p <* 0.01 compared with the control group, ## *p <* 0.01 DDP compared with DADS).

The MTT assay was used to detect the viability inhibitory effects of ECA109 cells and L02 cells incubated with various concentrations of DADS (10–60 μg/mL) for 24 h. DADS was dissolved in DMSO, and mixed with RPMI–1640 for experiments *in vitro*. The concentration of DMSO added to the medium of DADS was less than 0.01%. The medium with DMSO and in absence of DADS was used as the control group. In addition, we detected the difference of ECA109 cells incubated with DADS and *cis*-diammminedichloroplatinum (DDP) for 24 h. Our data showed that DADS obviously inhibited cell viability in a dose-dependent manner at concentrations of 10–60 μg/mL for 24 h (*p <* 0.05, Figure 1). Moreover, DADS had much lower cytotoxicity to L02 normal liver cells than ECA109 esophageal carcinoma cells (*p <* 0.05, Figure 1). Our data shows that DDP was more effective than DADS on ECA109 cells *in vitro* (*p <* 0.05, Figure 2); DDP was used as positive control for *in vivo* study.

### 2.2. DADS-Induced Apoptosis

**Figure 3:**
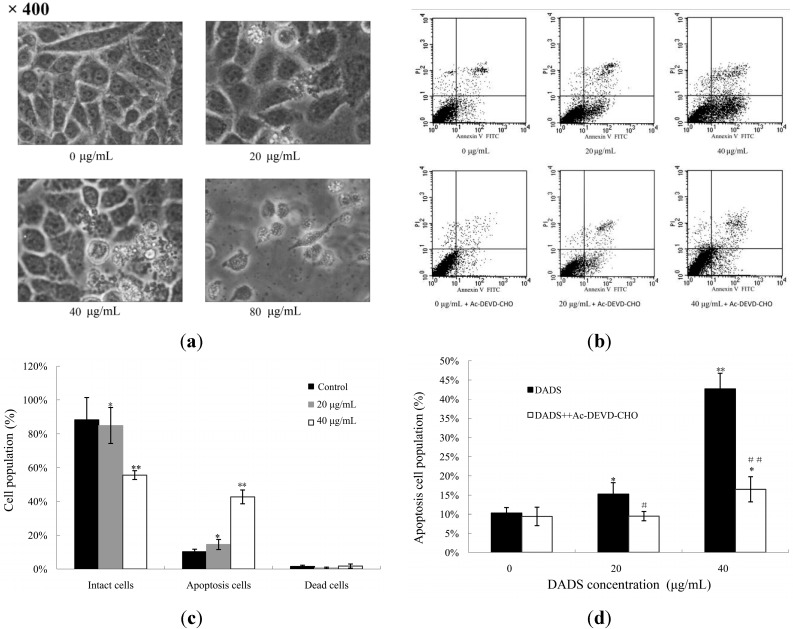
Apoptotic effects of DADS on ECA109 cells. (**a**) Morphology of ECA109 cells treated with different concentrations of DADS for 24 h and examined under phase contrast microscopy (×400); (**b**) DADS (0, 20 and 40 μg/mL) induced dose-dependent apoptosis in ECA109 cells. Ac-DEVD-CHO inhibited the apoptosis induced by DADS. Apoptosis was assessed using Annexin V-FITC/propidium iodide (PI) double staining; (**c**) Statistical analysis of the apoptosis rate of DADS; (**d**) Effects of Ac-DEVD-CHO against the apoptosis induced by DADS. Data are expressed as the mean ± SD from three independent experiments (* *p <* 0.05, ** *p <* 0.01 compared with the control group, # *p <* 0.05, ## *p <* 0.01 DADS + Ac-DEVD-CHO compared with DADS).

ECA109 cells were examined by phase contrast microscopy after being incubated with different concentrations of DADS (0, 20, 40, 80 μg/mL) for 24 h. The cells in the control group showed a typical intact appearance, whereas the DADS-treated cells displayed dose-dependent changes in cell shape. Membrane blebbing and formation of apoptotic bodies were found in 20 and 40 μg/mL DADS groups, while cellular shrinkage, poor adherence and floating shapes were found in the 80 μg/mL DADS group (Figure 3a).

As there were too many dead cells in the 80 μg/mL DADS group, we explored the apoptotic rate of ECA109 cells incubated with different concentrations of DADS (0, 20 and 40 μg/mL) for 24 h using Annexin V-FITC and propidium iodide (PI) staining and flow cytometry. Our data showed that the rate of apoptosis in the 0, 20 and 40 μg/mL DADS groups were (10.26 ± 1.45)%, (15.25 ± 2.99)% and (42.68 ± 4.08)% respectively. These results revealed that DADS induced the apoptosis of ECA109 cells in a dose-dependent manner (*p <* 0.05, Figure 3c). On the other hand, ECA109 cells were pretreated with caspase-3 inhibitor (Ac-DEVD-CHO) and then exposed to DADS for 24 h. Our results indicated that Ac-DEVD-CHO was able to protect ECA109 cells against DADS-induced apoptosis (*p <* 0.05, Figure 3d).

### 2.3. DADS Blocked the Growth of Xenograft Tumor

**Figure 4:**
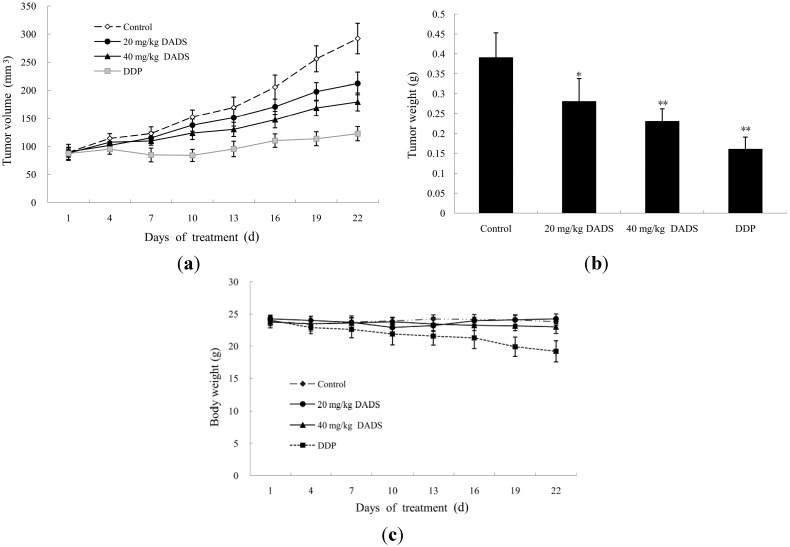
Effects of DADS on ECA109 xenograft tumor. Nude mice were implanted with ECA109 cells. After the xenograft tumors became palpable, intraperitoneal injections were made eight times every three days, including the negative control group, 20 mg/kg DADS group, 40 mg/kg DADS group and 2 mg/kg DDP positive control group. Effects of DADS on (**a**) tumor volume; (**b**) tumor weight; and (**c**) body weight were recorded. Data shown in **a**–**c** are the average of six mice in each group, and are expressed as the mean ± SD (* *p <* 0.05, ** *p <* 0.01 compared with the control group).

To evaluate the effect of DADS on the development of esophageal carcinoma *in vivo*, 5 × 10^6^ ECA109 cells were injected into the flanks of nude mice. After three weeks, all the mice developed palpable tumors. The total of twenty-four mice were randomly divided into four groups (*n =* 6 per group). DADS was dissolved in DMSO, and mixed with phosphate buffered saline (PBS) for intraperitoneal injections in nude mice. The concentration of DMSO added to the medium was less than 0.01%. The PBS medium with DMSO in absence of DADS was utilized as the negative control group. Injections of DADS at 20 and 40 mg/kg body weight were done in therapy groups. Injections of DDP at 2 mg/kg body wt were performed in the positive control group. These injections were made every three days, eight times. Compared with the negative control group, we observed significant difference of tumor weight in the 20 mg/kg DADS group (*p <* 0.05, Figure 4a), 40 mg/kg DADS group and DDP positive control group (*p <* 0.01, Figure 4a). At the end of the study, DADS decreased tumor volume from 292.02 ± 27.08 mm^3^ per mouse in the negative control group to 211.95 ± 20.14, 179.08 ± 15.95 and 122.64 ± 12.62 mm^3^ per mouse in 20 mg/kg DADS group (*p <* 0.05, Figure 4b), 40 mg/kg DADS group and DDP positive control group (*p <* 0.01, Figure 4b) respectively. In addition, there was no sign of possible changes of body weight in the DADS groups compared with the negative control group (*p >* 0.05, Figure 4c). However, the data showed that there was significant loss of body weight in DDP positive control group compared with the negative control group and DADS therapy groups (*p <* 0.05, Figure 4c).

### 2.4. DADS Inhibited Cell Proliferation and Induced Apoptosis in Xenograft Tumor

**Figure 5:**
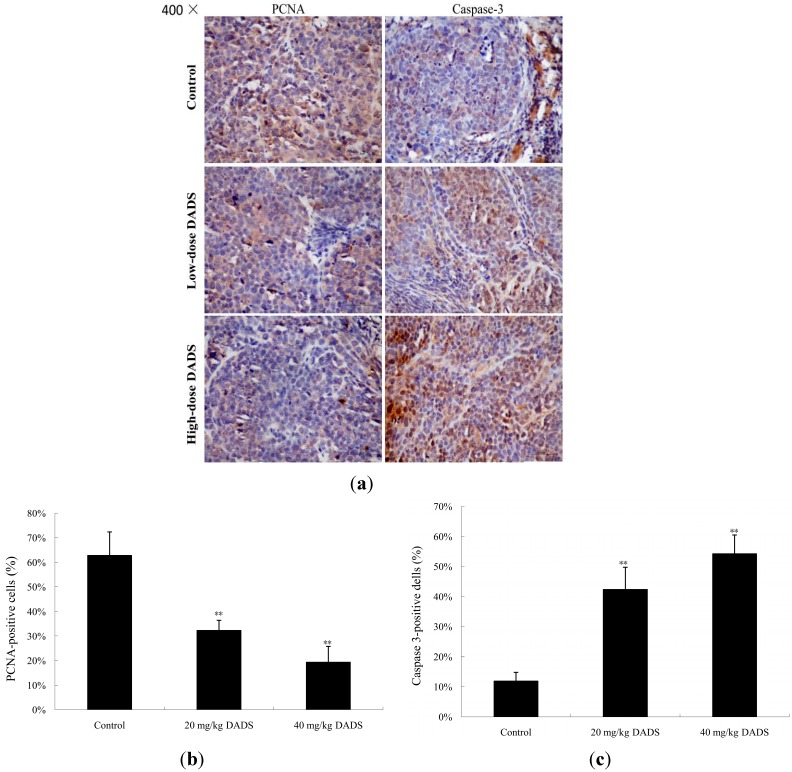
Anti-proliferative and pro-apoptotic effects of DADS on ECA109 xenograft tumors. Tumors from the negative control group, 20 mg/kg DADS group and 40 mg/kg DADS group were processed for immunohistochemical staining of proliferation cell nuclear antigen (PCNA) (**a**,**b**) and caspase-3 (**a**,**c**). A representative picture had been shown for each case. Immunoreactive cells (yellow to brown) in xenograft tumors were enumerated and analyzed by Image-Pro Plus analysis system (Media Cybernetics, Bethesda, MD, USA) linked to an Olympus microscope (Olympus Corporation, Center Valley, PA, USA). The total number of cells was presented from all the samples in each group of five randomly selected microscopic fields. The data are expressed as the mean ± SD (×400) (** *p <* 0.01 compared with the control group).

We analyzed the effects of DADS on ECA109 tumor proliferation and apoptosis by immunohistochemical staining of proliferation cell nuclear antigen (PCNA) and caspase-3 respectively. By microscopic observation, a larger number of PCNA immunoreactive cells was observed in the negative control group (PBS medium with DMSO and in absence of DADS was utilized for intraperitoneal injections in nude mice) than in the 20 and 40 mg/kg DADS groups (Figure 5a), which accounted for 62.79% ± 9.58%, 32.31% ± 4.13% and 19.29% ± 6.49% respectively (*p <* 0.01, Figure 5b). However, more caspase-3 immunoreactive cells were observed in the DADS groups than the negative control group (Figure 5a). The quantification of caspase-3 immunoreactive cells was 11.84% ± 2.99% in the control group, and was significantly increased to 42.37% ± 7.39% and 54.28% ± 6.25% in the 20 mg/kg DADS group and 40 mg/kg DADS group, respectively (*p <* 0.01, Figure 5c).

### 2.5. DADS Activated Mitochondria-Dependent Pathway and Up-Regulated Bax/Bcl-2 Ratio in Xenograft Tumors

**Figure 6:**
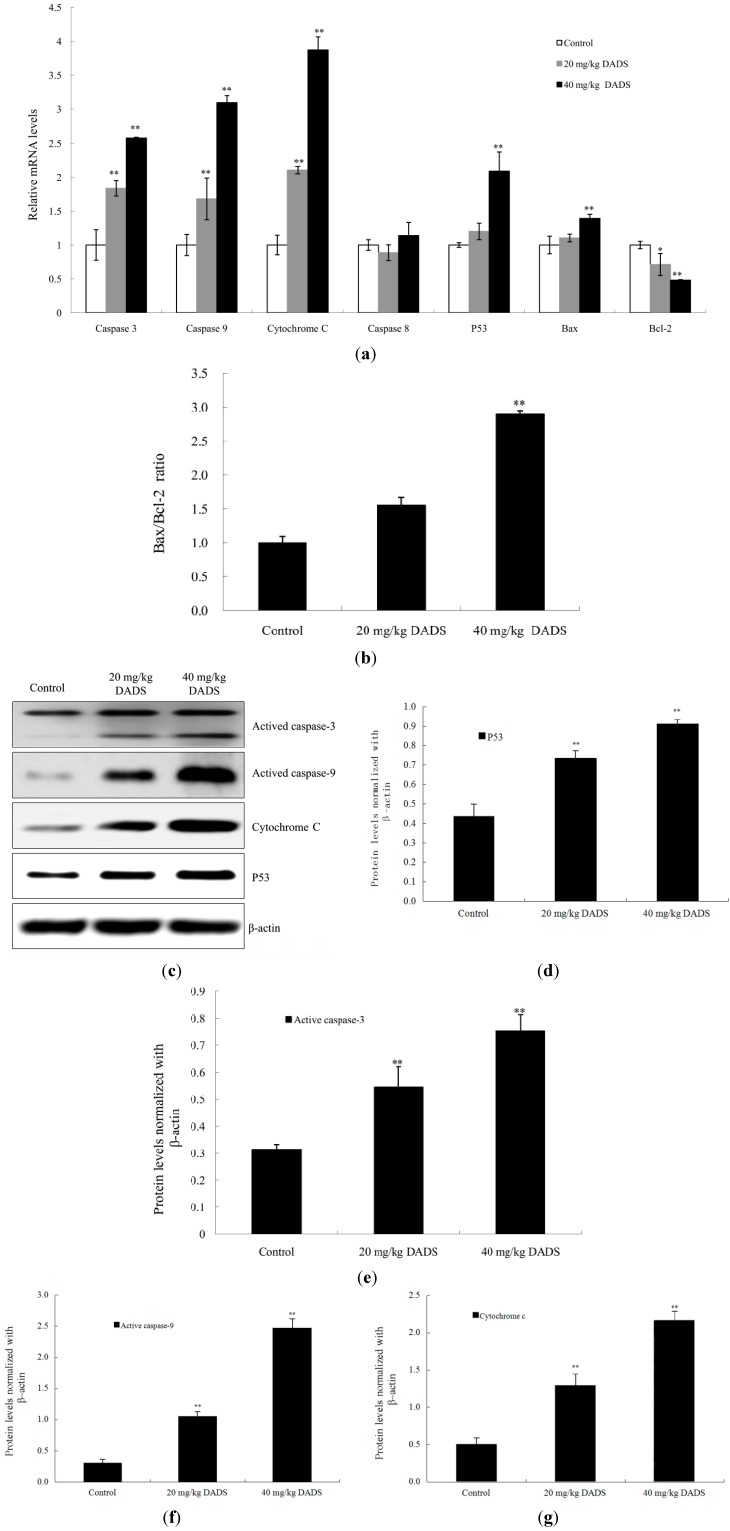
DADS activated mitochondria-dependent pathway and shift in Bax/Bcl-2 ratio in ECA109 xenograft tumors. The mRNA and protein expression levels of caspase-3, caspase-8, caspase-9, cytochrome C, Bax, Bcl-2 and p53 from the negative control group, 20 mg/kg DADS group and 40 mg/kg DADS group were assessed by qPCR and western blot. (**a**) The mRNA expressions of caspase-3, caspase-8, caspase-9, cytochrome C, Bax, Bcl-2 and p53; (**b**) Bax/Bcl-2 ratio; (**c**) Representative blots; Densitometric analysis was made on the expressions of p53 (7F5) (**d**); active caspase-3 (**e**); active caspase-9 (**f**) and cytochrome C (**g**). β-Actin was used as the loading control. Data are expressed as the mean ± SD from three independent experiments (* *p <* 0.05, ** *p <* 0.01 compared with the control group).

It is well known that mitochondria–mediated apoptosis involves the release of cytochrome C, as well as the activation of caspase-9 and caspase-3. Our results from qPCR indicated that the levels of cytochrome C, caspase-9 and caspase-3 were stimulated in DADS groups (*p <* 0.01, Figure 6a). Moreover, DADS significantly increased the expression levels of active caspase-3 and active caspase-9 in total proteins, as well as the expression levels of cytochrome C in cytoplasm proteins in 20 and 40 mg/kg DADS groups by western blot analysis (*p <* 0.01, Figure 6c,e–g). However, DADS did not affect caspase-8 expression levels (*p >* 0.05, Figure 6a).

On the other hand, Bcl–2 and Bax are major proteins of the mitochondria apoptosis pathway. Our results of qPCR showed that the expression levels of Bcl-2 decreased in the DADS groups (*p <* 0.05, Figure 6a), whereas the expression level of Bax increased only in the 40 mg/kg DADS group (*p <* 0.01, Figure 6a). Therefore, there was a significant up-regulation of the Bax/Bcl-2 ratio in the 40 mg/kg DADS group (*p <* 0.01, Figure 6b). In addition, the results of qPCR and Western blot suggested that DADS enhanced the expression levels of p53 in the 40 mg/kg group (*p <* 0.01, Figure 6a,e).

### 2.6. DADS Alters the RAF/MEK/ERK Pathway in Xenograft Tumors

**Figure 7:**
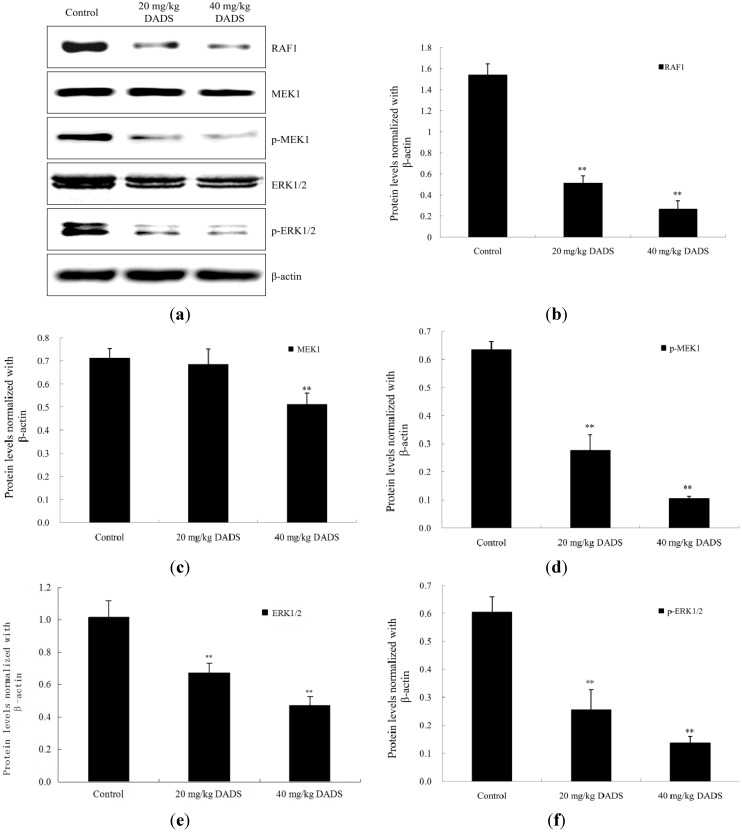
DADS down-regulated RAF/MEK/ERK pathway in ECA109 xenograft tumor. The protein expression levels of RAF1, MEK1, phosphor-MEK1 (p-MEK1), ERK1/2 and p-ERK1/2 from the negative control group, 20 mg/kg DADS group and 40 mg/kg DADS group were assessed by western blot analysis. (**a**) Representative blots. Densitometric analysis was made on the expressions of RAF1 (**b**); MEK1 (**c**); pMEK1 (**d**); ERK1/2 (**e**) and pERK1/2 (**f**). β-Actin was used as the loading control. Data are expressed as the mean ± SD from three independent experiments (** *p <* 0.01 compared with the control group).

Western blot analysis was used to evaluate the effects of DADS on the protein levels of RAF1, MEK1, phosphor-MEK1 (p-MEK1), ERK1/2 and p-ERK1/2 in ECA109 xenograft tumors. Our results showed a significant down-regulation of RAF1, p-MEK1, ERK1/2 and p-ERK1/2 protein expressions in 20 and 40 mg/kg DADS groups (*p <* 0.01, Figure 7b,d–f). The expression level of MEK1 decreased in 40 mg/kg DADS group (*p <* 0.01, Figure 7c). These results suggested that DADS had a profound effect on the apoptosis in ECA109 xenograft tumor, which might be correlated with the RAF/MEK/ERK pathway.

### 2.7. Discussion

DADS, a lipid-soluble organic compound isolated from garlic, is a potentially useful agent for cancer prevention and therapy, as it has the ability to reactivate the expression of genes in differentiation, cell cycle regulation, apoptosis and so on [10,12,15,16]. By using MTT assay, DADS showed a dose-dependent anti-viability effect on ECA109 cells *in vitro*. The viability inhibitory properties of DADS were attributed to its induction of apoptosis in human esophageal carcinoma ECA109 cells (Figure 1, Figure 2 and Figure 3). Moreover, we demonstrated that DADS had much lower cytotoxicity to normal liver cells L02 in comparison with ECA109 carcinoma cells (Figure 1). In addition, as the data showed that DDP had greater effect on ECA109 cells than DADS *in vitro* (Figure 2), we used DDP as the positive control in an *in vivo* study.

Studies of xenograft tumors represent well-established preclinical animal models for evaluating anticancer effects of test agents *in vivo*. Therefore, additional studies are warranted to check the effects of DADS by using animal models [11,14,17,18,19]. Researchers have injected DADS in nude mice at 30–200 mg/kg body wt intraperitoneally [17,18,19]. Consistent with these data, we selected suitable doses of DADS at 20 and 40 mg/kg body weight. Our study showed that DADS treatment inhibited the growth of ECA109 tumors in volume and weight at the two dosages used (Figure 4a,b). Importantly, intraperitoneal injections of DADS for eight times showed no apparent signs of toxicity. However, the data indicated that there was a significant loss of body weight in the DDP positive control group compared with the negative control group and DADS therapy groups (Figure 4c). Although we did not find a stronger anti-cancer effect of DADS compared to the traditional chemotherapy agent DDP, our study indicated that DADS could suppress the growth of ECA109 xenograft tumor effectively and had fewer side effects than DDP. Moreover, our data showed that DADS decreased the expression of tumor proliferation biomarker PCNA in the 20 and 40 mg/kg DADS groups (Figure 5a,b). Therefore, we suggest that DADS might be a promising candidate drug for esophageal carcinoma patients.

Apoptosis is programmed cell death that plays a major role during cancer treatment [20]. DADS has the ability to induce apoptosis of some human tumor cells. However, the apoptosis induced by DADS involves different apoptotic genes and proteins depending on the cell types of different tumors [15,16]. The effects of DADS on esophageal carcinoma are still unclear, especially *in vivo*. Our study proved the apoptosis effect of DADS on ECA109 cells by using several methods. First, under light microscope, we observed morphology changes of apoptosis, such as membrane blebbing and formation of apoptotic bodies in the 20 and 40 μg/mL DADS groups, and cellular shrinkage, poor adherence and floating shapes in the 80 μg/mL DADS group (Figure 3a). Second, we detected the apoptosis rate by the double-staining of Annexin V-FITC and PI. Our results revealed that DADS induced the apoptosis of ECA109 cells in a dose-dependent manner (Figure 3b,c). In addition, the study indicated that DADS might inhibit the viability of ECA109 cells by promoting apoptosis.

Apoptosis signals are controlled by two distinct pathways, including the extrinsic pathway (death receptor pathway) and the intrinsic pathway (mitochondrial pathway) [21]. Caspases are cysteine proteases that play pivotal roles in apoptosis. Fourteen caspases have been identified based on their functions, which are divided into two groups (initiator caspases and effector caspases) [22]. Initiator caspases (caspase-2, -8, -9, and -10) are activated as a result of protein complex formation [23]. In the death receptor pathway, the activation of the death receptor leads to initiation of a caspase cascade by caspase-8. The mitochondrial pathway is characterized by the release of cytochrome C from mitochondria and the activation of a caspase cascade through caspase-9 [24]. Caspase-3, -6 and -7 function as executioners. Caspase-3 is a crucial executor that cleaves various substrates related to apoptosis [23,25]. Most of the apoptosis procedures induced by DADS are executed by caspase-3. However, some kinds of apoptosis are independent with caspase-3 [26,27,28,29]. In order to prove the apoptosis of DADS on ECA109 cells, we studied caspase-3 by qPCR and western blot *in vitro* and *in vivo*, as well as by immunohistochemical assay of xenograft tumor *in vivo*. Our results showed that the apoptosis of DADS was caspase-dependent, since caspase-3 was active during this procedure (Figure 5a,c and Figure 6a,c,e) and that the pre–treatment with caspase-3 inhibitor (Ac-DEVD-CHO) could dramatically block the apoptosis induced by DADS (Figure 3b,d). Besides, many agents can activate cellular apoptotic programs through the mitochondria pathway, which triggers changes in regulatory factors [10]. Some soluble proteins including cytochrome C and Apaf-1, which can combine with caspase-9 to form the apoptosome, are released from the mitochondrial intermembrane space, and subsequently initiate an apoptosis cascade [30]. The mechanisms of apoptosis induced by DADS are different in various cells of different carcinomas. DADS induces apoptosis and promotes the activities of caspase-8 and caspase-9 in human colon cancer cell line (COLO 205) [26]. However, in the present study, our results of qPCR and western blot indicated that the activations of caspase-9 and caspase-3 as well as the release of cytochrome C from mitochondria to cytoplasm were stimulated in DADS groups (Figure 6a,c,f,g), which were involved in the typical mitochondrial apoptosis pathway [24]. Hence, these data suggested that the apoptosis effect induced by DADS on ECA109 cells was caspase-dependent *in vitro* as well as *in vivo*. In addition, our results indicated that one central mechanism of the apoptosis inducing by DADS in esophageal xenograft tumor was the activation of the mitochondrial pathway, but not the death receptor pathway.

The activation of caspases has a close relationship with the transduction of apoptosis signalling. P53 is known to inhibit cell proliferation and induce caspase-mediated apoptosis. Bax is a key target of p53 transcription factor in apoptosis [31]. Bcl-2, an antiapoptotic protein, is able to bind and inactivate Bax to modulate tumor cells responding apoptosis [32]. P53 and the Bax/Bcl-2 ratio play important roles in the apoptotic process. The molecular mechanisms of DADS on various carcinomas are still a matter of debate. DADS could up-regulate the p53 level to take part in the apoptotic processes of melanoma in the B16F-10 cell line and human cervical cancer Ca Ski cell line [33,34]. However, the effect of DADS is independent of p53 activity in the osteosarcoma Saos-2 cell line [35]. Moreover, p53 is increased in the colon cancer COLO 205 cell line, and is decreased in the colon cancer SW480 cell line by DADS treatment [26,36]. These experiments have indicated that DADS has various peculiarities in the treatment of different carcinomas. Moreover, even different cell lines of the same carcinoma are probably not alike under DADS treatment. P53 has been found to mutate in 83% esophageal squamous cell carcinomas [2], while p53 is highly expressed in the ECA109 cell line [37]. In the present study, our results of qPCR and Western blot suggested that DADS could enhance p53 expression levels in the two dosages of DADS groups, especially in the 40 mg/kg DADS group (Figure 6a,c,d). By qPCR assay, we found that mRNA expression levels of Bcl-2 were down-regulated in the two DADS groups, whereas the expression level of Bax was up-regulated in the 40 mg/kg DADS group (Figure 6a). Moreover, our data indicated the significant increase of the Bax/Bcl-2 ratio in the 40 mg/kg DADS group (Figure 6b), leading to the pro-apoptosis of ECA109 cells. Based on these observations, our study showed that the up-regulations of p53 expression levels and the Bax/Bcl-2 ratio were involved in DADS-induced cell death.

The RAF/mitogen-activated protein kinase/extracellular signal-regulated kinase (RAF/MEK/ERK) pathway plays a prominent role in the regulation of cell growth [38]. The RAF/MEK/ERK pathway can be affected by the activation of p53 [39]. ERK is a downstream component of conserved signaling module activated by the serine/threonine kinase, RAF. RAF can be recruited to the cellular membrane and activate MEK, which phosphorylates and activates ERK in turn [38]. ERK phosphorylation may control transcription by targeting several different regulators such as transcription factors and histone proteins, which result in proliferation, differentiation and protection against apoptosis [40]. DADS has been shown to be a histone deacetylase (HDAC) inhibitor, which has the ability to affect the growth and survival of tumor cells [12]. Although some studies have indicated that DADS inhibits the phosphorylation of ERK1/2 during the apoptotic processes of the human leukemia HL-60 cell line and human colon cancer COLO 205 cell line [16,41], other studies have shown that DADS activates ERK1/2 in human non-small cell lung cancer H1299 cell line and human nasopharyngeal carcinoma CNE2 cell line [15,42]. Moreover, DADS has no influence on ERK1/2 in apoptosis of the human prostate carcinoma DU145 cell line [10]. In the present study, our western blot results showed that the expression levels of RAF1, phosphor-MEK1 (p-MEK1), ERK1/2 and p-ERK1/2 were inhibited in both the 20 and 40 mg/kg DADS groups (Figure 7a,b,d–f), and that the expression level of MEK1 was down-regulated in the 40 mg/kg DADS group (Figure 7a,c). Based on these findings, it seemed that the down-regulation of the RAF/MEK/ERK signaling pathway contributed to the apoptosis induced by DADS in the ECA109 xenograft tumor. Moreover, the RAF/MEK/ERK pathway might be a valuable therapy target for esophageal carcinoma in the future.

## 3. Experimental Section

### 3.1. Reagents and Antibodies

ECA109 human esophageal carcinoma cell line and L02 human normal liver cell line were purchased from the Chinese Academy of Shanghai Institute of Cell Biology. DADS and DDP were purchased from Sigma–Aldrich Chemical Company, St. Louis, MO, USA. DMSO, MTT, propidium iodide (PI), phosphate buffered saline (PBS) were purchased from Sigma–Aldrich. Fetal bovine serums (FBS), RPMI-1640 medium and 0.25% trypsin were purchased from Hyclone Company, Logan, UT, USA. Trizol was purchased from Invitrogen, Grand Island, NY, USA. Annexin V-FITC apoptosis kit was purchased from Roche Technology Company, Branchburg, NJ, USA. PrimeScript™ RT Master Mix kit and SYBR^®^ Premix Ex Taq II kit were purchased from Takara Technology Company, Sakado-shi, Saitama, JAPAN. Enhanced chemiluminescence (ECL) kit was purchased from Amersham Life Science, Arlington Heights, UK. Materials and chemicals used for electrophoresis were obtained from Bio-Rad Laboratories, Hercules, CA, USA. ProteoJET cytoplasmic Protein Extraction kit was purchased from Fermentas, Pittsburgh, PA, USA. Antibodies to RAF1, MEK1, phosphor-MEK1 (p-MEK1), ERK1/2, phosphor-ERK1/2 (p-ERK1/2), p53 (7F5), caspase-3, active caspase-3, caspase-9, active caspase-9, cytochrome C and proliferation cell nuclear antigen (PCNA) were purchased from Cell Signaling Technology Company, Danvers, MA, USA. The β-actin antibody was purchased from Santa Cruz Biotechnology, Dallas, TX, USA. Horseradish peroxidase- (HRP-) coupled goat anti-mouse IgG and anti-rabbit IgG (secondary antibody) were purchased from Santa Cruz Biotechnology.

### 3.2. Cell Culture

Human esophageal carcinoma cell line ECA109 and human normal liver cell line L02 were cultured in RPMI-1640 supplemented with 10% FBS under standard culture condition (37 °C, 95% humidified air and 5% CO_2_). Exponentially growing ECA109 cells were used for all assays.

### 3.3. Cell Viability Assay

Cell viability was tested by MTT assay. ECA109 cells and L02 cells were seeded in 96-well plates at 1 × 10^4^ cells/well and incubated with DADS or DDP (10–60 μg/mL) in five replicates for 24 h. Cells treated with PBS with DMSO and in absence of DADS were used as the negative control. After the incubation, 5 mg/mL MTT reagent (20 μL) was added into each well, followed by the addition of 150 μL DMSO. The plates were measured at 570 nm (A570) by spectrophotometer. The percentage of cell viability inhibition rate was calculated according to the following formula:

Cell viability (%) = 1 − [(A570 (control) − A570 (sample)/A570 (control)] × 100%
(1)

### 3.4. Apoptosis Assay

The apoptosis rate was determined by using an Annexin V-FITC detection kit. ECA109 cells of 5 × 10^5^ were incubated with different concentrations of DADS (0, 20, 40 μg/mL) and 10 μM caspase-3 inhibitor (Ac-DEVD-CHO) with DADS for 24 h. The cells were resuspended in 100 μL binding buffer at 1 × 10^6^ cells/mL, and incubated with 5 μL Annexin V-FITC and 10 μL PI for 15 min in the dark. Then, these cells were added into 400 μL binding buffer and measured by flow cytometer. Apoptosis was analyzed by Cell Quest software (BD Biosciences, San Jose, CA, USA).

### 3.5. Xenograft Tumor Assay in Vivo

Female BALB/c nude mice (4–6 weeks old) were purchased from Beijing Experimental Animal Center. These mice were handled according to the Guidelines for the Care and Use of Laboratory Animals with the approval of the Medical Ethics Committee of the Second Affiliated Hospital of Xi’an Jiaotong University (ID: 2012120, 01 November 2012). Exponentially growing ECA109 cells of 5 × 10^6^ were suspended in 200 µL RPMI–1640 and injected subcutaneously into the flank of each mouse. After growth for three weeks, all the mice developed palpable tumors. Twenty-four mice were randomly divided into four groups (*n =* 6 per group). The intraperitoneal injections of PBS with DMSO and in absence of DADS as the negative control group, DADS at 20 and 40 mg/kg body weight as therapy groups, DDP at 2 mg/kg body weight as the positive control group were made every three days for eight times. Tumors were monitored every three days by measuring length and width with a vernier caliper (tumor volume = 0.5 × L × W^2^). All the mice were sacrificed three days after the last injection and the xenograft tumors were observed and measured.

### 3.6. Immunohistochemical Staining

Tumor samples were formalin-fixed and paraffin-embedded. Paraffin blocks were cut serially at 5 µm thick. The sections were incubated with a specific primary antibody, such as PCNA (1:100) and caspase-3 (1:100), for 1 h at 37 °C followed by the overnight incubation at 4 °C in humidity chamber. Then, they were incubated with an appropriate biotinylated secondary antibody (1:200–1:400) followed by conjugated horseradish peroxidase–streptavidin and 3,3'-diaminobenzidine working solution, and then counterstained with hematoxylin. Immunoreactive cells (yellow to brown) were enumerated and analyzed by Media Cybernetics Image-Pro Plus analysis system linked to Olympus microscope. The total number of cells was presented from five randomly selected microscopic fields of every sample in each group (×400).

### 3.7. Quantitative Real-Time PCR

Total RNA was extracted from each tumor sample by Trizol method, and the quantity of RNA was assessed by spectrophotometry. The cDNA was obtained by reverse transcription with 1 µg total RNA by using PrimeScript™ RT Master Mix kit. qPCR was made with SYBR Premix Ex Taq™ II Perfect Real Time kit. All the reactions were performed on ABI qPCR System. The individual value was normalized by the loading control, β-actin. The mRNA expression was expressed as fold, and comparative cycle threshold (*C*_t_) method was used to study the relative quantification of gene expression. Sequences of gene primers were listed in Table 1.

### 3.8. Western Blot Assay

ECA109 xenograft tumors of the negative control group and the two DADS groups were washed with cold PBS and homogenized in RIPA lysis buffer. The homogenates were centrifuged at 10,000× *g* for 10 min at 4 °C, and supernatants were collected as total proteins. Moreover, cytoplasmic proteins were prepared by using ProteoJET cytoplasmic Protein Extraction kit according to the manufacturer’s instruction.

The proteins were separated by 8%–12% sodium dodecyl sulfate-polyacrylamide gel electrophoresis (SDS-PAGE), transferred to polyvinylidene difluoride (PVDF) membrane, and probed with a specific primary antibody, such as RAF1 (1:1000), MEK1 (1:1000), p-MEK1 (1:500), ERK1/2 (1:1000), p-ERK1/2 (1:300), caspase-3 (1:1000), active caspase-3 (1:500), caspase-9 (1:1000), active caspase-9 (1:500), cytochrome C, p53 (7F5) (1:1000) and β-actin (1:1000), followed by an appropriate peroxidase–conjugated secondary antibody (1:5000–1:10,000). The individual value was normalized by the loading control, β-actin. Antigen-antibody complex signals were visualized using BeyoECL Plus (Amersham Life Science). In addition, densitometric analysis was performed by Image J software (National Institutes of Health, Bethesda, MD, USA).

### 3.9. Quantification and Statistic Analysis

Quantitative data were expressed as the mean ± SD from at least three independent experiments. The two-tailed student’s *t*-test was performed for paired samples, and one-way ANOVA or two-factor factorial ANOVA was used for multiple groups. The *p* value less than 0.05 was considered significant. Statistical analyses were performed by SPSS 17.0 statistics software (IBM, Armonk, NY, USA).

## 4. Conclusions

In summary, the present study demonstrated that DADS suppresses esophageal tumors without any apparent signs of toxicity, which is in agreement with a strong increase of apoptosis both *in vitro* and *in vivo*. DADS inhibits ECA109 tumor proliferation through the down-regulation of PCNA. Moreover, DADS induces apoptosis by activating the mitochondria-dependent pathway executed by caspase-3, increasing p53 expression level and the Bax/Bcl-2 ratio, and down-regulating the RAF/MEK/ERK pathway in ECA109 xenograft tumors. Overall, our studies of DADS in cell culture and animal models indicate that DADS is a potentially effective and safe anti-cancer agent for esophageal carcinoma treatment.

## References

[1] Liu R., Peng Y., Li X., Wang Y., Pan E., Guo W., Pu Y., Yin L. (2013). Identification of plasma metabolomic profiling for diagnosis of esophageal squamous-cell carcinoma using an UPLC/TOF/MS platform. Int. J. Mol. Sci..

[2] Song Y., Li L., Ou Y., Gao Z., Li E., Li X., Zhang W., Wang J., Xu L., Zhou Y. (2014). Identification of genomic alterations in oesophageal squamous cell cancer. Nature.

[3] Cui X., Zhao Z., Liu D., Guo T., Li S., Hu J., Liu C., Yang L., Cao Y., Jiang J. (2014). Inactivation of miR-34a by aberrant CpG methylation in Kazakh patients with esophageal carcinoma. J. Exp. Clin. Cancer Res..

[4] Shen Z.T., Wu X.H., Li B., Shen J.S., Wang Z., Li J., Zhu X.X. (2013). Nedaplatin concurrent with three-dimensional conformal radiotherapy for treatment of locally advanced esophageal carcinoma. World J. Gastroenterol..

[5] Yeruva L., Elegbede J.A., Carper S.W. (2008). Methyl jasmonate decreases membrane fluidity and induces apoptosis through tumor necrosis factor receptor 1 in breast cancer cells. Anti-Cancer Drugs.

[6] Eswar K., Venkateshbabu N., Rajeswari K., Kandaswamy D. (2013). Dentinal tubule disinfection with 2% chlorhexidine, garlic extract, and calcium hydroxide against *Enterococcus faecalis* by using real-time polymerase chain reaction: *In vitro* study. J. Conserv. Dent..

[7] Truong D., Hindmarsh W., O’Brien P.J. (2009). The molecular mechanisms of diallyl disulfide and diallyl sulfide induced hepatocyte cytotoxicity. Chem. Biol. Interact..

[8] Lee I.C., Kim S.H., Baek H.S., Moon C., Kim S.H., Kim Y.B., Yun W.K., Kim H.C., Kim J.C. (2013). Protective effects of diallyl disulfide on carbon tetrachloride-induced hepatotoxicity through activation of Nrf2. Environ. Toxicol..

[9] Alam M., Zubair S., Farazuddin M., Ahmad E., Khan A., Zia Q., Malik A., Mohammad O. (2013). Development, characterization and efficacy of niosomal diallyl disulfide in treatment of disseminated murine candidiasis. Nanomed. Nanotechnol. Biol. Med..

[10] Shin D.Y., Kim G.Y., Lee J.H., Choi B.T., Yoo Y.H., Choi Y.H. (2012). Apoptosis induction of human prostate carcinoma DU145 cells by diallyl disulfide via modulation of JNK and PI3K/AKT signaling pathways. Int. J. Mol. Sci..

[11] Tang H., Kong Y., Guo J., Tang Y., Xie X., Yang L., Su Q., Xie X. (2013). Diallyl disulfide suppresses proliferation and induces apoptosis in human gastric cancer through Wnt-1 signaling pathway by up-regulation of miR-200b and miR-22. Cancer Lett..

[12] Myzak M.C., Dashwood R.H. (2006). Histone deacetylases as targets for dietary cancer preventive agents: Lessons learned with butyrate, diallyl disulfide, and sulforaphane. Curr. Drug Targets.

[13] Jun Z., Suzuki M., Xiao J., Wen J., Talbot S.G., Li G.C., Xu M. (2009). Comparative effects of natural and synthetic diallyl disulfide on apoptosis of human breast-cancer MCF-7 cells. Biotechnol. Appl. Biochem..

[14] Lai K.C., Kuo C.L., Ho H.C., Yang J.S., Ma C.Y., Lu H.F., Huang H.Y., Chueh F.S., Yu C.C., Chung J.G. (2012). Diallyl sulfide, diallyl disulfide and diallyl trisulfide affect drug resistant gene expression in colo 205 human colon cancer cells *in vitro* and *in vivo*. Phytomed. Int. J. Phytother. Phytopharmacol..

[15] Hui C., Jun W., Ya L.N., Ming X. (2008). Effect of Allium sativum (garlic) diallyl disulfide (DADS) on human non-small cell lung carcinoma H1299 cells. Trop. Biomed..

[16] Tan H., Ling H., He J., Yi L., Zhou J., Lin M., Su Q. (2008). Inhibition of ERK and activation of p38 are involved in diallyl disulfide induced apoptosis of leukemia HL-60 cells. Arch. Pharm. Res..

[17] Liao Q.J., Su J., Zhou X.T., Tang H.L., Song Y., Su Q. (2007). Inhibitory effect of diallyl disulfide on proliferation of human colon cancer cell line SW480 in nude mice. Chin. J. Cancer.

[18] Xiang S.L., Xiao X.L., Ling H., Liao Q.J., Zhou X.T., Dong L., Su Q. (2005). Antitumor effect of diallyl disulfide on human gastric cancer MGC803 cells xenograft in nude mice. Chin. J. Cancer.

[19] Zhao J., Huang W.G., He J., Tan H., Liao Q.J., Su Q. (2006). Diallyl disulfide suppresses growth of HL-60 cell through increasing histone acetylation and p21WAF1 expression *in vivo* and *in vitro*. Acta Pharmacol. Sin..

[20] Haneji T., Hirashima K., Teramachi J., Morimoto H. (2013). Okadaic acid activates the PKR pathway and induces apoptosis through PKR stimulation in MG63 osteoblast-like cells. Int. J. Oncol..

[21] Ozoren N., El-Deiry W.S. (2003). Cell surface death receptor signaling in normal and cancer cells. Semin. Cancer Biol..

[22] Jin Z., El-Deiry W.S. (2005). Overview of cell death signaling pathways. Cancer Biol. Ther..

[23] Olsson M., Zhivotovsky B. (2011). Caspases and cancer. Cell Death Differ..

[24] Thomas S.A., Vasudevan S., Thamkachy R., Lekshmi S.U., Santhoshkumar T.R., Rajasekharan K.N., Sengupta S. (2013). Upregulation of DR5 receptor by the diaminothiazole DAT1 [4-amino-5-benzoyl-2-(4-methoxy phenyl amino) thiazole] triggers an independent extrinsic pathway of apoptosis in colon cancer cells with compromised pro and antiapoptotic proteins. Apoptosis Int. J. Program. Cell Death.

[25] Zheng T.S., Hunot S., Kuida K., Momoi T., Srinivasan A., Nicholson D.W., Lazebnik Y., Flavell R.A. (2000). Deficiency in caspase-9 or caspase-3 induces compensatory caspase activation. Nat. Med..

[26] Yang J.S., Chen G.W., Hsia T.C., Ho H.C., Ho C.C., Lin M.W., Lin S.S., Yeh R.D., Ip S.W., Lu H.F. (2009). Diallyl disulfide induces apoptosis in human colon cancer cell line (COLO 205) through the induction of reactive oxygen species, endoplasmic reticulum stress, caspases casade and mitochondrial-dependent pathways. Food Chem. Toxicol..

[27] Nagaraj N.S., Anilakumar K.R., Singh O.V. (2010). Diallyl disulfide causes caspase-dependent apoptosis in human cancer cells through a Bax-triggered mitochondrial pathway. J. Nutr. Biochem..

[28] Altonsy M.O., Habib T.N., Andrews S.C. (2012). Diallyl disulfide-induced apoptosis in a breast-cancer cell line (MCF-7) may be caused by inhibition of histone deacetylation. Nutr. Cancer.

[29] Gayathri R., Gunadharini D.N., Arunkumar A., Senthilkumar K., Krishnamoorthy G., Banudevi S., Vignesh R.C., Arunakaran J. (2009). Effects of diallyl disulfide (DADS) on expression of apoptosis associated proteins in androgen independent human prostate cancer cells (PC-3). Mol. Cell. Biochem..

[30] Sun K.W., Ma Y.Y., Guan T.P., Xia Y.J., Shao C.M., Chen L.G., Ren Y.J., Yao H.B., Yang Q., He X.J. (2012). Oridonin induces apoptosis in gastric cancer through Apaf-1, cytochrome c and caspase-3 signaling pathway. World J. Gastroenterol..

[31] Liu J., Qin C.K., Lv W., Zhao Q., Qin C.Y. (2013). OSU-03012, a non-Cox inhibiting celecoxib derivative, induces apoptosis of human esophageal carcinoma cells through a p53/Bax/cytochrome c/caspase-9-dependent pathway. Anti-Cancer Drugs.

[32] Produit-Zengaffinen N., Pournaras C.J., Schorderet D.F. (2009). Retinal ischemia-induced apoptosis is associated with alteration in Bax and Bcl-x(L) expression rather than modifications in Bak and Bcl-2. Mol. Vis..

[33] Pratheeshkumar P., Thejass P., Kutan G. (2010). Diallyl disulfide induces caspase-dependent apoptosis via mitochondria-mediated intrinsic pathway in B16F-10 melanoma cells by up-regulating p53, caspase-3 and down-regulating pro-inflammatory cytokines and nuclear factor-kappabeta-mediated Bcl-2 activation. J. Environ. Pathol. Toxicol. Oncol..

[34] Lin Y.T., Yang J.S., Lin S.Y., Tan T.W., Ho C.C., Hsia T.C., Chiu T.H., Yu C.S., Lu H.F., Weng Y.S. (2008). Diallyl disulfide (DADS) induces apoptosis in human cervical cancer Ca Ski cells via reactive oxygen species and Ca^2+^-dependent mitochondria-dependent pathway. Anticancer Res..

[35] Masuelli L., Marzocchella L., Focaccetti C., Tresoldi I., Palumbo C., Izzi V., Benvenuto M., Fantini M., Lista F., Tarantino U. (2012). Resveratrol and diallyl disulfide enhance curcumin-induced sarcoma cell apoptosis. Front. Biosci..

[36] Liao Q.J., Su J., He J., Song Y., Tang H.L., Su Q. (2009). Effect of diallyl disulfide on cell cycle arrest of human colon cancer SW480 cells. Chin. J. Cancer.

[37] Wang T.T., Wang S.K., Huang G.L., Sun G.J. (2012). Luteolin induced-growth inhibition and apoptosis of human esophageal squamous carcinoma cell line Eca109 cells *in vitro*. Asian Pac. J. Cancer Prev..

[38] Ciccarelli A., Giustetto M. (2014). Role of ERK signaling in activity-dependent modifications of histone proteins. Neuropharmacology.

[39] Bhalla S., Evens A.M., Dai B., Prachand S., Gordon L.I., Gartenhaus R.B. (2011). The novel anti-MEK small molecule AZD6244 induces BIM-dependent and AKT-independent apoptosis in diffuse large B-cell lymphoma. Blood.

[40] Lv C., Sun W., Sun H., Wei S., Chen R., Wang B., Huang C. (2013). Asperolide A, a marine-derived tetranorditerpenoid, induces G2/M arrest in human NCI-H460 lung carcinoma cells, is mediated by p53–p21 stabilization and modulated by Ras/Raf/MEK/ERK signaling pathway. Mar. Drugs.

[41] Lai K.C., Hsu S.C., Kuo C.L., Yang J.S., Ma C.Y., Lu H.F., Tang N.Y., Hsia T.C., Ho H.C., Chung J.G. (2013). Diallyl sulfide, diallyl disulfide, and diallyl trisulfide inhibit migration and invasion in human colon cancer colo 205 cells through the inhibition of matrix metalloproteinase-2, -7, and -9 expressions. Environ. Toxicol..

[42] Zhang Y.W., Wen J., Xiao J.B., Talbot S.G., Li G.C., Xu M. (2006). Induction of apoptosis and transient increase of phosphorylated MAPKs by diallyl disulfide treatment in human nasopharyngeal carcinoma CNE2 cells. Arch. Pharm. Res..

